# Secure Drone Network Edge Service Architecture Guaranteed by DAG-Based Blockchain for Flying Automation under 5G

**DOI:** 10.3390/s20216209

**Published:** 2020-10-30

**Authors:** Ying Gao, Yangliang Liu, Quansi Wen, Hongliang Lin, Yijian Chen

**Affiliations:** School of Computer Science and Engineering, South China University of Technology, Guangzhou 510641, China; gaoying@scut.edu.cn (Y.G.); 201821034188@mail.scut.edu.cn (Y.L.); cslhlin@mail.scut.edu.cn (H.L.); csyjchan@mail.scut.edu.cn (Y.C.)

**Keywords:** drones, flying automation, edge service, DAG-based blockchain, 5G mobile network

## Abstract

With the development of the Internet of Things (IoT), the number of drones, as a consumer-level IoT device, is rapidly increasing. The existence of a large number of drones increases the risk of misoperation during manual control. Therefore, it has become an inevitable trend to realize drone flying automation. Drone flying automation mainly relies on massive drone applications and services as well as third-party service providers, which not only complicate the drone network service environment but also raise some security and privacy issues. To address these challenges, this article proposes an innovative architecture called Secure Drone Network Edge Service (SDNES), which integrates edge computing and blockchain into the drone network to provide real-time and reliable network services for drones. To design a feasible and rational SDNES architecture, we first consider the real-time performance and apply edge computing technology in it to provide low-latency edge services for drones under 5G mobile network. We use DAG-based blockchain to guarantee the security and reliability of the drone network service environment and effectively avoid malicious behaviors. In order to illustrate the feasibility of this architecture, we design and implement a specific service case named Drone Collision Avoidance Navigation Service based on SDNES. Finally, a simulation experiment for the specific service case and a series of other performance-related experiments were carried out to verify the feasibility and rationality of our proposed architecture. The experimental results demonstrate that SDNES is a promising architecture to assist and accelerate drone flying automation.

## 1. Introduction

Due to the rapid development of the Internet of Things (IoT), IoT devices are not only applied to industrial fields such as smart manufacturing, logistics, and energy trading, but they are also penetrating into people’s daily life such as with smart healthcare, smart cities, and smart homes [1]. Among them, drones, also known as Unmanned Ariel Vehicles (UAVs), have become one of the most prevalent IoT devices in recent years because of its high flexibility, high adaptability, low cost, and small size [2,3,4].

Drones have changed from military-level IoT devices to consumer-level IoT devices and have been applied to increasing civil and commercial fields including parcel delivery, aerial photography, geological survey, traffic measurement [5], rescue search [6], and so on. This tendency leads to a drastic increase in the number of drones. It is predicted that the number of drones will reach 7 million by 2020 [7]. However, current drone flying relies primarily on continuous control by users within the line of sight (LOS) [8]. Such a flying mode will not be able to adapt to the rapid increase in the number of drones for the following three reasons: (1) non-professional operators can lead to misoperation of drones, which greatly increases the risk of accidents such as drone collisions or intrusions into the no-fly zone. For example, there were incidents in which drones flew onto the premises of the White House or caused threats to commercial aviation pilots at airports [9]. (2) A Drone user cannot control multiple drones at the same time to complete non-cooperative tasks. (3) It is unreasonable for users to pay attention to the drone at all times during a task. Therefore, it is an inevitable trend to realize drone flying automation. Note that drone flying automation not only refers to automatic flying, but it also includes automatic execution of preset tasks and solving problems encountered during tasks by the drone itself without any manual intervention.

To meet the demand of drone flying automation, user drones will require increasing applications and the corresponding network services. However, to the best of our knowledge, few studies have been conducted on the secure service architecture for drone networks. The majority of research uses drones as aids to solve other network problems. For example, in [8], drones are used to assist vehicular networks to provide ubiquitous connections for vehicles. Authors in [10] leverage drones to provide Voice over WiFi (VoWiFi) service to a set of ground users confined in an open area. In [11], drones are deployed to assist establishing a multi-connectivity system to support mission-critical machine-type communications (mcMTC) in IoT. Since no systematic architecture of drone network service has been proposed, it slows down the generation of services and applications, and it further limits the realization of drone flying automation. Based on this situation, a complete drone network service architecture is urgently needed to assist and accelerate realizing drone flying automation. Therefore, how to design a secure, reliable and real-time drone network service architecture is the focus of this article.

In some traditional networks, network services are provided by centralized cloud servers, which means the cloud servers need to handle all of the service requests and data processing. This undoubtedly causes a huge computing burden to the cloud servers. The centralized cloud servers are usually deployed at remote centers, which forms a long transmission distance between the users and servers, leading to high service latency. Such a service paradigm is not suitable for drone network since drone applications for some special scenarios are latency-critical, such as in navigation. To address above challenges, a new paradigm called edge computing [12] is applied to provide edge service. By offloading some computation tasks to the edge of the network, it will greatly improve the data processing efficiency and reduce the load on the cloud. Authors in [13] utilize edge computing to migrate the deep learning process from cloud servers to edge nodes, and the result shows that the time consumption of edge calculation is 38% that of center computing, which is reduced by 62%. Moreover, since edge service are deployed in the proximity of users, it can provide users with faster service response [14]. For drone flying automation, in addition to network services, reliable network communication is also necessary. The fifth-generation (5G) mobile network technology provides ultra-reliable and affordable broadband access everywhere to a massive number of IoT devices [15]. Therefore, 5G technology can offer a massive number of drones at higher speed, with low latency, and ubiquitous communication connectivity [16].

The security of drone network services is also an issue that should be taken into consideration because there will be a large amount of data exchange and data storage requirements during the drone flying automation process. If some sensitive or important data cannot be well protected, it will cause various potential security threats such as privacy leaks and compromised data integrity [17]. However, as many untrusted third-party cloud service providers join the drone network to provide edge services, the drone network service environment will become complex and unreliable. For example, some malicious behaviors may occur, including collecting user privacy information and tampering with user data. Therefore, some secure privacy and data integrity protection mechanisms need to be introduced into the drone network. Fortunately, blockchain technology provides feasible solutions to these issues [18,19,20], since it provides a reliable consensus in a distrusted environment without trust. Especially in [21], the authors present a blockchain-based security scheme in 5G drone networks to ensure privacy preservation during communications between drones, which demonstrates the feasibility of leveraging blockchain to facilitate privacy preservation in the drone network. Because of its anonymity, encryption, traceability, and non-tamperability, blockchain can provide good protection for user privacy security and data integrity. However, traditional blockchains such as Bitcoin [22] and Ethereum [23] are limited by the throughput and hardware resources of devices due to their proof-of-work (PoW) or proof-of-stake (PoS) consensus mechanisms. They are not suitable for IoT. Therefore, blockchain based on the new consensus mechanism of directed acyclic graph (DAG) is born. The DAG-based blockchain has also been proved to be better for IoT than the traditional blockchains [24,25]. The authors in [26] also show that blockchain can be used to ensure secure edge services under complex industrial networks.

**Figure 1:**
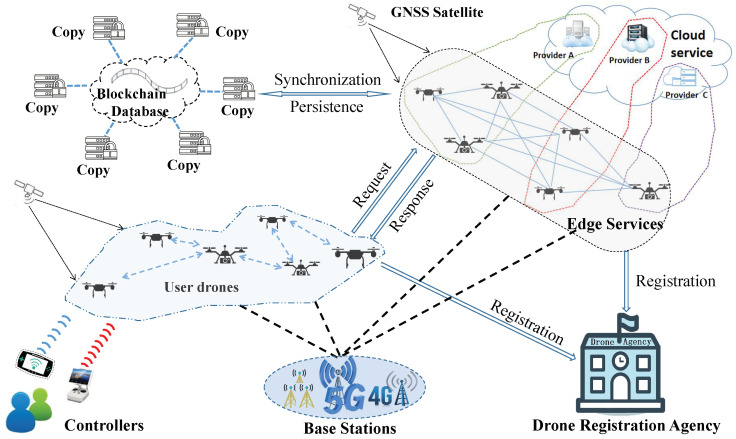
Illustration of secure drone network edge service scenario.

To sum up, this article sought to contribute providing the secure drone network service architecture foundation for the realization of drone flying automation. Figure 1 gives an illustration of our architecture scenario. Our contributions can be summarized as follows:

- This article proposes an innovative architecture called Secure Drone Network Edge Service (SDNES) that aims to provide network services for drones and thus assists to realize drone flying automation.
- Considering the architecture’s performance and security needs, this article integrates edge computing technology and blockchain into the architecture to enhance the real-time performance of drone network services under the 5G mobile network, guaranteeing the security and reliability of the service environment, and effectively avoiding malicious behaviors.
- In order to illustrate the feasibility of this architecture, we design and implement a specific service case named Drone Collision Avoidance Navigation Service based on SDNES.
- Finally, a simulation experiment for the service case and a series of other related experiments were carried out to verify the feasibility and rationality of our proposed architecture.

The remainder of this article is organized as follows. Section 2 gives an overview of DAG-based blockchian. Section 3 explains how the SDNES architecture works. Then, the implementation principle and advantages of Drone Collision Avoidance Navigation Service are described in Section 4. Next, in Section 5, a simulation experiment and a series of other performance-related experiments were carried out and discussed. Our final conclusion and some open issues are given in Section 7.

## 2. Overview of DAG-Based Blockchain

**Figure 2:**
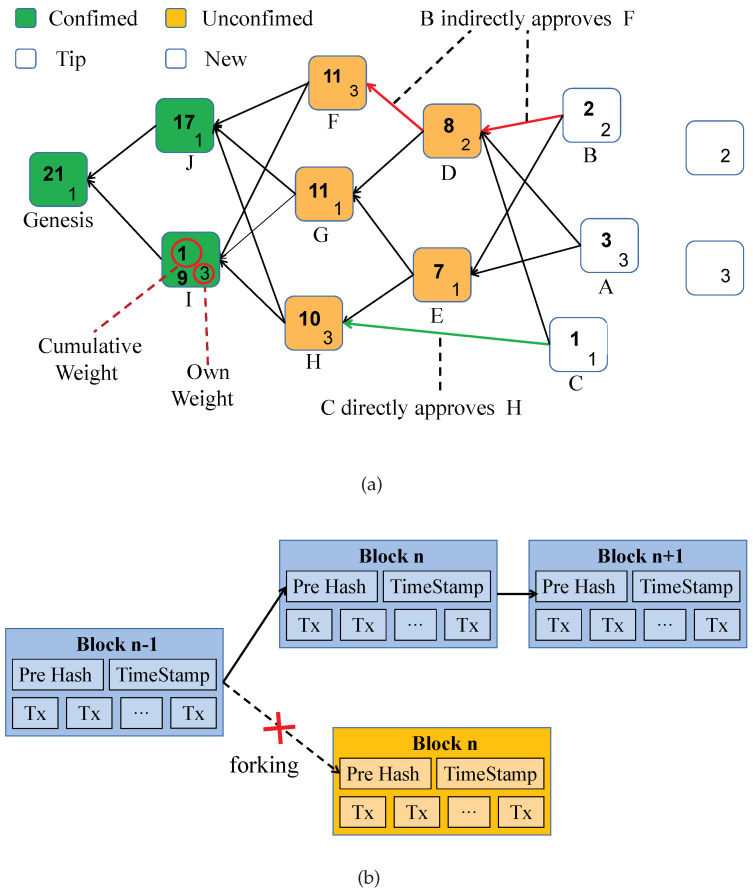
Blockchain data structure comparison: (**a**) data structure of Tangle and (**b**) data structure of a traditional blockchain.

DAG-based blockchains such as Tangle [27] aim to improve the performance of traditional blockchains by applying a new data structure and consensus mechanism. As shown in Figure 2, the data structure of Tangle is based on a directed acyclic graph, while traditional blockchain is based on a linked list. The main difference between the blockchains with these two data structures is whether they allow forking. In the Linked-list based blockchain, forking is not allowed. It happens when multiple blocks are generated in the same block generation cycle, and only one block will be written into the blockchain as an effective block. However, in the DAG-based blockchain, forking is allowed, which enables DAG-based blockchain to process transactions in parallel, greatly improving the throughput. It is well known that Bitcoin’s transaction throughput is 7 TPS and Ethereum’s is 20 to 30 TPS [24]. An efficient and compacted DAG-based blockchain protocol (CoDAG) proposed in [28] shows that CoDAG achieves a throughput 164 times that of Bitcoin’s and 77 times that of Ethererum’s throughput, respectively. When it comes to the consensus mechanism, traditional blockchains are usually based on the PoW or PoS. Blockchain nodes reach consensus through competition of computing power or stake, which results in higher requirements on the equipment performance of nodes. However, in DAG-based blockchains, the newly generated transaction only needs to select two tips to verify, then add the hashes of these two tips to the unit of this transaction to approve. There is no competition but mutual supervision in DAG-based blockchains.

Therefore, this article adopts Tangle, a DAG-based blockchain, in our SDNES architecture.

**Some Related Terms**

Here, the following blockchain-related terms that will be used in our architecture are introduced:

- **Asymmetric Encryption:** A pair of keys including the public key and the private key can be generated by the asymmetric encryption algorithm. The public key is public to other users in the blockchain, and the private key is kept by the owner himself. Data encrypted with a public key can only be decrypted with the corresponding private key.
- **Digital Signature:** A digital signature is essentially a ciphertext generated by the signer using the private key to encrypt a message. The recipient verifies the legality of the signer by comparing whether the message obtained by decrypting the ciphertext using the public key of the signer is consistent with the message sent by the signer.
- **Address:** The blockchain uses the public key as both the user’s communication address and identity identification.
- **Hash Value:** A hash value is a fixed-length character string that is calculated from original data through the hash function. Based on the same hash function, the hash value calculated from the same original data must be unique, and it is almost impossible for different original data to get the same hash value. Therefore, if two hash values are different, then the original data of these two hash values are also different.

## 3. SDNES Architecture

### 3.1. Functions and Design Details

**Figure 3:**
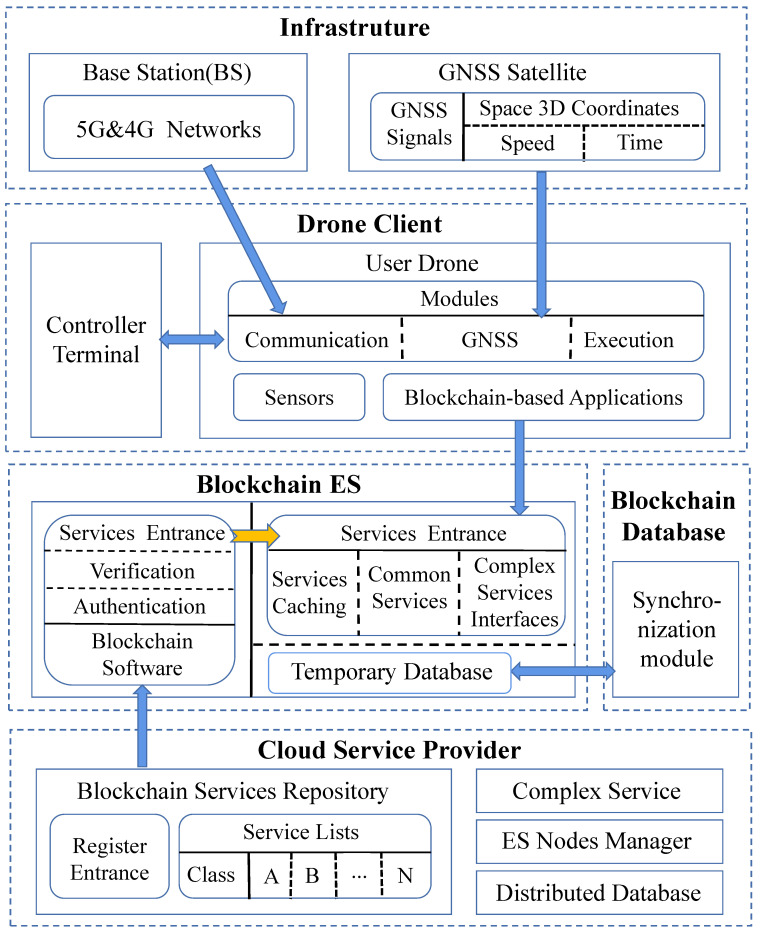
Architecture of SDNES.

As shown in Figure 3, our proposed architecture consists of the following six parts:

**Drone Registration Agency (DRA):** DRA is a government drone management agency whose main responsibility is to register the real identity information of drone users and issue the electronic certificates for drones. The users’ real identity information, drone device information, and drone certificates will be stored in the DRA’s local user identity database. At the same time, the DRA binds the certificates and the public keys of drones and records them in the blockchain, which are used to verify the legality of the drones.

**Cloud Service Provider (CSP):** CSP refers to various third-party cloud service providers. Their main function is to create a variety of drone network services. After creating new services, they pack these services as service plugins and upload them to the service repository for registration and sharing. Some complex services that consume a large amount of computing and storage resources are provided directly by CSPs, and only the interfaces are registered to the service repository. For the storage of some data of user drones, the distributed database is well deployed in this part. Furthermore, since most drones in Blockchain ES (explained later) are provided by CSPs, this part also provides an ES node manager to manage and maintain these drones.

**Blockchain ES:** This part is the core part of SDNES. It effectively integrates the edge service and blockchain into the drone network. Specifically, some commercial drones equipped with high performance are deployed into the drone network and serve as nodes for both the edge service and blockchain. Here, the blockchain plays four main roles: (1) It performs node authentication tasks. When a new drone applies to join Blockchain ES, it broadcasts its certificate and digital signature to existing nodes in Blockchain ES. Existing nodes will check whether the certificate exists in the blockchain and verify the legality of the signature with the public key corresponding to the certificate. Only after the majority nodes have verified that the new drone is legal, the new drone can serve as a node of Blockchain ES, but can leave at any time. (2) As a unified service resource management platform, blockchain also serves as a services entrance by integrating the services registered in the service repository into edge service nodes. Besides providing some ordinary services, interfaces of complex services and service caching are also provided. When edge service nodes provide normal edge services, the blockchain may do some additional operations. For example, for some data storage service requests, the blockchain is used to record the hash values of the data sent by the user drones. Some service records are also recorded in the blockchain. (3) It performs verification tasks of some service responses and data integrity. Some service responses require joint verification of nodes to ensure the correctness of responses. For data integrity verification, because the hash value of the original data is recorded in the blockchain, this can be used to verify whether the data has been modified. (4) Blockchain is also used for credit evaluation tasks. Through user drone service feedback and mutual evaluation of nodes while performing the verification, credit evaluation of edge nodes and edge services can be realized, and the credit evaluation results are recorded in the blockchain. Blockchain ES also provides a temporary database for storage of blockchain data and other data of user drones.

**Blockchain Database (BD):** BD is used to persistently store data recorded in the blockchain, including drone certificates, hash values, service records, credit evaluation results of edge nodes and edge services, and so on. These data records are transparent and can be queried by users and drones. Because the drones in Blockchain ES may leave at any time for various reasons such as a lack of power or service failure, their data storage is temporary. In order to avoid data loss, BD synchronizes the blockchain data in the Blockchain ES periodically. For the drones that want to join Blockchain ES, besides updating services from the service repository, it is also necessary to download some historical records from BD to ensure normal query and verification services.

**Drone Client:** In this part, user controllers and user drones are included. The user controllers only need to perform part of the control and configuration works such as task setting, recovery landing point setting, and so on. The various applications that run on the user drones to assist flying automation are also blockchain-based. Therefore, user drones become clients of the blockchain system. They can send service requests to the Blockchain ES. Especially, according to the location and load of the Blockchain ES nodes, with credit evaluation results of edge nodes and edge services, the blockchain software can choose the most suitable nodes to respond to service requests. After completing the service responses, user drones can automatically evaluate the services. Users can also perform artificial evaluations based on service records.

**Infrastructure:** This part mainly includes Global Navigation Satellite System (GNSS) and Base Stations (BS). GNSS is used to provide drones with 3D coordinates, speed, and time information, whereas BS is to provide wireless mobile network coverage for drones as well as controllers communications. The BS mainly comprises 5G base stations, but some 4G base stations are still reserved for communication compatibility.

### 3.2. Advantages Analysis of SDNES

- **Secure Drone Service Environment Guarantee**
  Our SDNES uses two mechanisms to guarantee secure drone service environment. First, the node authentication mechanism is used to achieve the legality verification of all drones applying to join the Blockchain ES. In addition, credit evaluation of edge nodes and edge services in the drone network is achieved through a credit evaluation mechanism, and the evaluation results are recorded in the blockchain. The edge nodes or edge services with poor credit evaluation results may be used by fewer and fewer user drones, so the credit evaluation mechanism can achieve the purpose of public supervision of edge nodes and edge services. Note that authentication and evaluation do not need to rely on any third-party institutions, but reach consensus through blockchain nodes, making them more authentic and reliable.
- **Privacy Protection**
  The blockchain uses asymmetric encryption methods to protect data privacy. When user drones need to exchange data, they can use the recipient’s public key to encrypt the data. For some important data that needs to be stored in the CSP’s distributed database, user drones can also use their own public keys for encryption and then send the encrypted data to Blockchain ES. In addition, because the public key does not contain any user information, using the public key as the identity and communication address of the user drones can meet the anonymity requirement and thus protect the user’s identity privacy.
- **Data Integrity Verification**
  In SDNES, when the Blockchain ES receives the data storage requests from the user drones, it first calculates the hash values of original data and stores them in the blockchain, then transmits original data to CSP. Based on the characteristics of the hash value, the integrity of the data can be verified by comparing the hash value generated from the data stored in the CSP’s distributed database with the hash value in the blockchain. If the match fails, the data integrity is damaged. Data integrity verification can effectively detect malicious data modification behavior of CSP. With the use of a distributed database and some specific mechanisms, data that have been tampered with can be recovered.
- **Resistance to Quantum Computing Attack**
  Although the current asymmetric encryption algorithms have high security, with the continuous development of quantum computing, it is possible to calculate the private key corresponding to the public key in polynomial time [29]. Therefore, if the same public key is used all the time, the corresponding private key may be leaked. In order to address this issue, Tangle generates a unique seed (a character string) for each user. By using it in combination with two other parameters, security level and index, the seed can easily generate different levels of addresses as well as the corresponding private keys, where the security level is a number between 1 and 3 and the index is a number from 1 to 9,007,199,254,740,991. Therefore, a seed can have an almost unlimited number of addresses (9${}^{57}$), which gives user drones the ability to use different addresses for each service request. After that, the used addresses will become spent addresses, which means that they can continue to receive service responses but no longer be used for requests. In addition, if the private key corresponding to the public key bound to the drone certificate is calculated by quantum calculation, the drone certificate may be used maliciously by other drones. To prevent this from happening, the drone can also periodically replace the public key bound to the certificate. Specifically, the drone can use the digital signature of the private key corresponding to the original public key to prove the legality of the identity, thereby completing the binding of the certificate and new public key. Therefore, this property of Tangle can greatly resist quantum computing attacks and thus improve the security of SDNES architecture.
- **Real-time Performance**
  A hierarchical edge service structure is adopted in SDNES to speed up the edge service response. Besides providing some ordinary services, interfaces of complex services and service caching for some general services are also provided in Blockchain ES. Among them, ordinary services refer to services that consume less resources and can be provided directly by the edge nodes themselves. For complex services, because CSP usually has more resources than edge nodes, it is faster for CSP to directly respond to complex service requests. For those general services that do not rely on user parameter information and will not change in a short time, Blockchain ES caches them for fast response. Such a hierarchical edge service structure enables edge nodes to use different response strategies according to different service requests, thereby improving the real-time performance of services.

## 4. Drone Collision Avoidance Navigation Service

**Figure 4:**
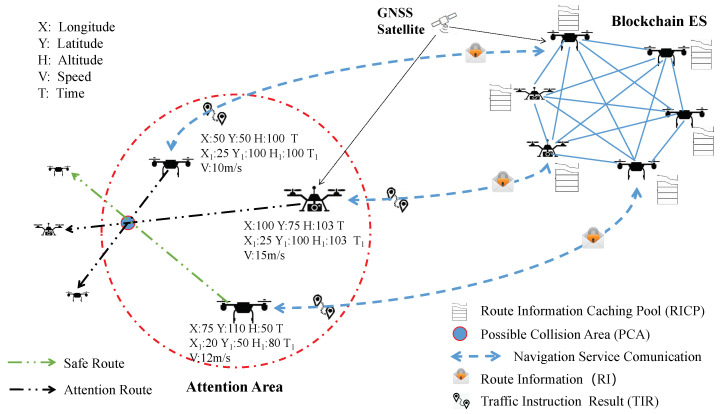
Drone collision avoidance navigation service. The drone cluster in the upper right corner constitutes Blockchain ES, and the drones in the red circle on the left represent user drones.

In order to illustrate the feasibility of our proposed SDNES architecture, we design a specific service case called Drone Collision Avoidance Navigation Service. Figure 4 graphically summarizes the scenario for this service case, whose purpose is to avoid collision between user drones during autonomous flying. This section describes the implementation principle and advantages of this service, and the specific simulation experiment will be described in the next section.

**Figure 5:**
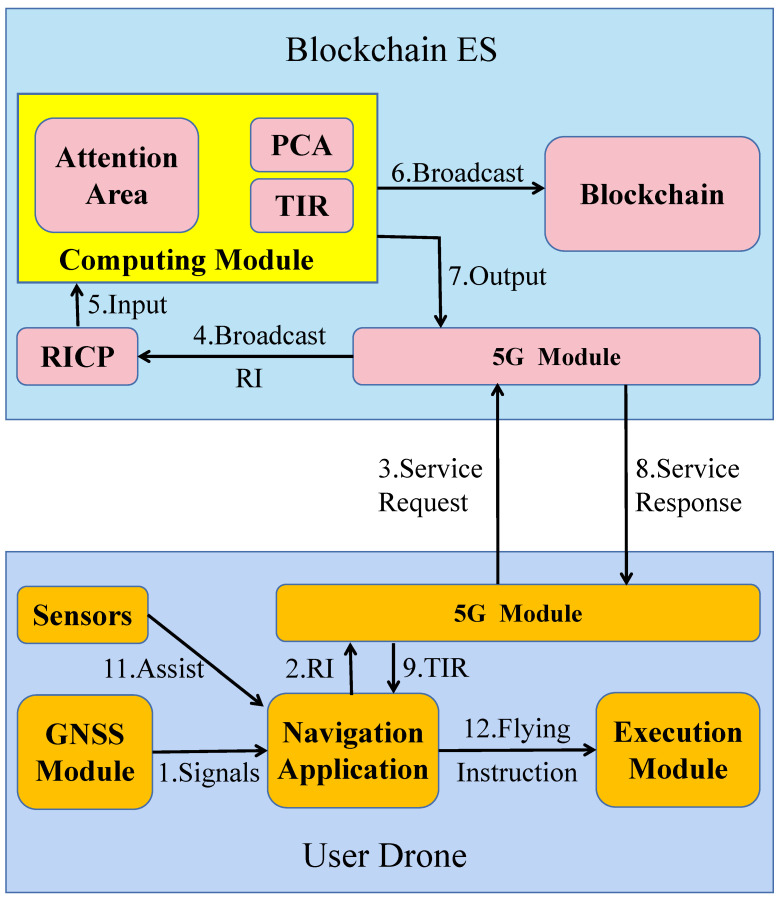
Service implementation process.

**Implementation Principle:** In order to achieve collision avoidance navigation, user drones should interact with the Blockchain ES periodically. As shown in Figure 5, by running the navigation application, the user drones get the GNSS signals from the GNSS module, including 3D coordinates, speed, and time information. Then, the application packs this information into a data packet, called route information (RI). RI can be represented by a vector (X, Y, H, T, X${}_{1}$, Y${}_{1}$, H${}_{1}$, T${}_{1}$, V), where X, Y, H, T, and V represent the longitude, latitude, altitude, time, and speed of the user drones, respectively. Therefore, RIs can represent the route information of user drones from T to T${}_{1}$. Then, the drone clients initiate service requests to send the RIs to the Blockchain ES through the 5G module. When the Blockchain ES nodes receive the service requests, they extract the RIs and addresses of drone clients and broadcast them to Blockchain ES. All nodes in the Blockchain ES maintain a route information caching pool (RICP), which is used to temporarily store RIs. RICP periodically cleans up invalid RIs whose T${}_{1}$ has become historical time. In order to detect possible collision areas (PCAs), RIs are used as the input for the calculation module. Through specific algorithms, each node continuously detects whether the relative spatial position of some user drones is within a certain range, namely the Attention Area. When Attention Areas appear, it is necessary to analyze RIs of drones in the Attention Area and calculate to detect if there are PCAs. If they exist, nodes will calculate the traffic instruction results (TIRs) and broadcast them to other nodes of Blockchain ES for verification. TIRs and the identity of the nodes that calculate the TIRs will be recorded in the blockchain. In case of drone traffic accidents, this information will be used as evidence for penalty, compensation, and credit evaluation. TIRs are also sent back to the corresponding addresses of the drone clients through the 5G module. The clients then generate flying instructions with the assistance of the sensors, and they transmit them to the execution module so that the user drones can be alerted in advance and take measures, including detour or hover, to avoid possible collisions.

**Route Privacy Protection:** In the process of this service, user drones need to send RIs to the Blockchain ES periodically. If the RIs of user drones can be continuously tracked by Blockchain ES nodes through the same address, the route privacy will be leaked. This kind of design is unreasonable and sometimes even dangerous when the drone performs some secret tasks. In this service case, this issue can be well addressed. Specifically, for each service request, the user drones use different addresses to send RIs, which makes the Blockchain ES nodes unable to track the routes of specific user drones, but the TIRs can still be directly sent back to the corresponding drone clients through the spent addresses. Therefore, this service can protect the route privacy of user drones from being leaked while realizing the collision avoidance navigation of drones.

## 5. Experiments and Discussion

In this section, we carried out a series of experiments, including the simulation experiment of the service case described in the previous section and other experiments, to test the performance of our proposed SDNES architecture. Then, experimental results are discussed and analyzed.

The experimental environment and related configurations are as follows:

- One Cloud Server: We deployed a server with 8 Intel Xeon CPU @2.2GHz processors, 8 GB RAM, and running Ubuntu18.04 OS as the Cloud Server.
- Two Edge Servers: We deployed two servers with 4 Intel Xeon CPU @2.2GHz processors, 4 GB RAM, and running Ubuntu18.04 OS as the Blockchain ES nodes.
- DAG-based Blockchain: We adopted Tangle to provide the blockchain service. The specific node software of Tangle we used is Hornet in version 0.5.3.

### Simulation Experiment

We implemented the prototype of Drone Collision Avoidance Navigation Service to simulate the collision avoidance navigation of drones. Specifically, we ran nine (set randomly, but not too large) client threads on a Win10 Operating System with 8 CPUs and 16G RAM to simulate nine user drones. The program running in each thread is the drone anti-collision application. We also set some parameters in advance for the application according to the actual situation of the drone. For example, we set the maximum flight speed of the user drone to 20 m/s, and we set the diameter of the attention area to 100 m. The time interval T${}_{\mathrm{interval}}$ for the user drone to send RIs varied according to the flight speed and the number of drones in unit space. In particular, every time the user drone changed its flight direction, it resent the RI to the Blockchain ES. T${}_{\mathrm{interval}}$ is calculated by the following formula

(1) $$T_{\mathrm{interval}}\;=\;T_{\mathrm{base}}\times \mathrm{Min}\left(1,\frac{V_{\mathrm{base}}}{v}\right)\times \mathrm{Min}\left(1,\frac{N_{\mathrm{base}}}{n}\right)$$

where T_base_ equals 2 s, V_base_ equals 10 m/s, and N_base_ equals 100, which represent the basic time interval, speed, and number of drones in the basic unit space, respectively. In addition, v represents the current flying speed of a drone, and n represents the number of drones in current unit space, which can be obtained from the server. In our simulation experiment, since v is less than 20 m/s and n is less than 100, the user drone sends RIs every 1–2 s.

**Figure 6:**
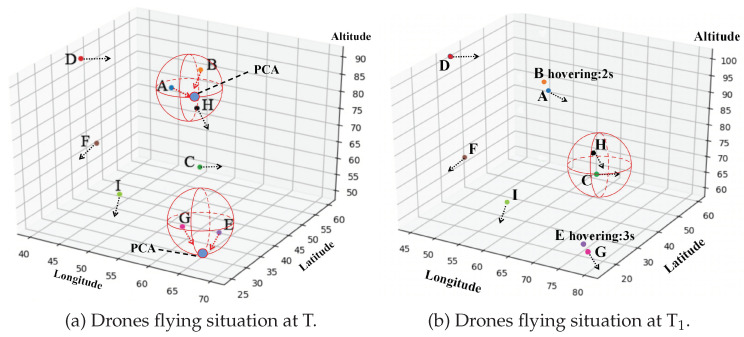
Experiment results. In (**a**) and (**b**), each point represents a user drone, lettered from A to I, and the red 3D spheres represents Attention Areas. The axes are scaled to a certain extent, and each unit length represents 10 m of the actual space.

Figure 6a,b shows the drone flying situations at two moments T and T${}_{1}$ during a simulation. At time T, Blockchain ES detected two Attention Areas, and it was calculated that there were two PCAs between A and B, E, and G. Then, Blockchain ES sent different TIRs to A, B, E, and G respectively. After receiving TIRs, each user drone took a different flying action according to TIRs at time T${}_{1}$. For example, B and E hovered in the air to wait for A and G flying through involved PCAs, then continued to fly along the original routes. At the same time, although Blockchain ES detected that the relative spatial position of C and H entered into an Attention Area, it was found that PCA did not exist after analyzing their RIs.

In each simulation experiment, the drones could successfully avoid possible collision, which verified the feasibility of the specific service case we designed, and further proves the feasibility of SDNES architecture.

## 6. Performance Experiments

In addition to the simulation experiment, we also conducted a comparative experiment and two other related experiments to verify the rationality of our proposed architecture design. In this way, the performance of our proposed architecture can also be tested in the current experimental environment and configurations.

**Figure 7:**
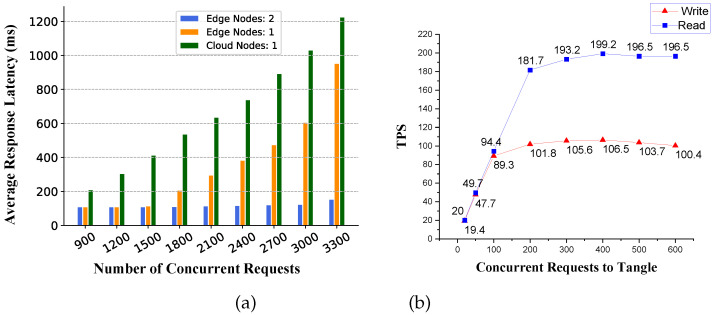
Throughput of SDNES architecture: (**a**) Real-time performance of SDNES and (**b**) TPS of Tangle${}_{1}$.

**Figure 8:**
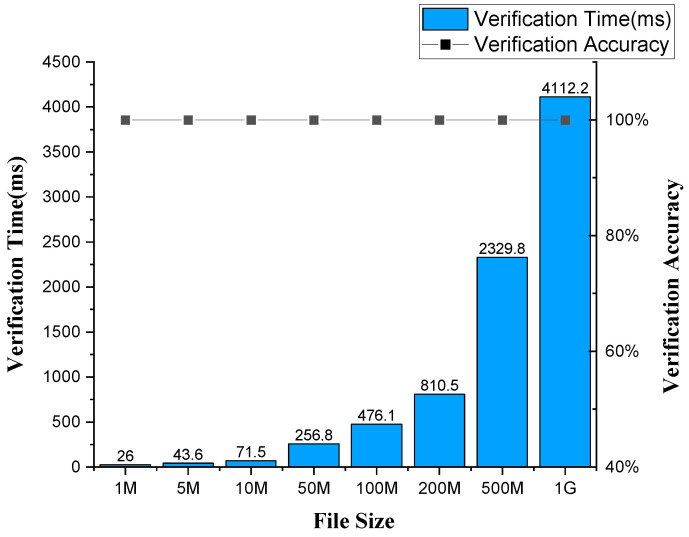
Data integrity verification.

(1) Real-time Performance of SDNES: In order to test whether our proposed architecture has high real-time performance, we tested it under high-concurrency pressure. The experimental result is shown in Figure 7, and it shows that when the number of concurrent requests was less than 3000, the average response latency of all requests was less than 1 s, which meets the real-time requirements of most applications. By comparing the average response latency of the cloud service and edge service, we find that the edge service greatly reduced network latency. It can also be seen from the figure that the average response time changed significantly by changing the number of edge nodes. The results show that with a higher number of concurrent requests, the higher the number of edge nodes, the lower the average response latency. Therefore, by deploying multiple nodes at the edge, the real-time requirements of the architecture can be thoroughly met.

(2) TPS of Tangle: Because our architecture uses DAG-based blockchain, in order to verify that the performance of this form of blockchain is indeed better than Bitcoin and Ethereum, we conducted high-concurrency read and write tests on a single node in the Tangle network. The experimental results shown in Figure 7b show that, under the current configurations, the maximum numbers of read and write transactions per second for a single Tangle node were about 102 and 196, respectively, which are much higher than the throughput of Bitcoin and Ethereum. By increasing the number of nodes or improving hardware performance, the overall read-write speed of the Tangle network can be further improved.

(3) Data Integrity Verification: As an important technology to ensure security of SDNES, one of the core functions of blockchain is to ensure the integrity of data generated by user drones, so as to effectively prevent data from being maliciously modified. This function is very useful for safe storage of drone files or data. In order to test the performance of data integrity verification, we verified the integrity of files of different sizes from 1M to 1G. Specifically, we randomly generated eight types of files of different sizes, as shown in Figure 8. Each type of file contained 100 files with the same size. First, we calculated all the file hashes and uploaded them to the blockchain. Then, we made some arbitrary changes to the files in each type and marked the modified files. We then use the data integrity verification service developed based on SDNES to verify the file integrity. The experimental results show that all the modified files could be detected accurately, and all the unmodified files passed the integrity verification. The accuracy of file integrity verification reached 100%. In addition, in the experiment, we also calculated the average verification time for each type of file. The results show that the verification can be completed within 1 s for files within 200M, and only a little more than 4 s for 1G files. This experiment proves that the application of blockchain in our architecture can guarantee data integrity. In addition, blockchain is also effective for privacy protection, which will be explored in our subsequent research.

## 7. Conclusions and Open Issues

This article has proposed a SDNES architecture that aims to provide network services for drones and thus assists to realize drone flying automation. In SDNES architecture, it integrates edge c and DAG-based blockchain to significantly ensure real-time performance and security of the services. Specifically, a hierarchical edge service structure is adopted in SDNES to speed up service response under the 5G mobile network. Based on the blockchain, this article introduces node authentication and credit evaluation mechanisms to guarantee the security and reliability of the service environment. Privacy protection, data integrity verification, and resistance to quantum computing attacks are also achieved in our architecture. This article also designs and implements a specific Drone Collision Avoidance Navigation Service case and conducts a simulation experiment. By conducting the simulation study, it proves that it is feasible to use SDNES architecture to design and implement reliable services. Finally, several performance-related experiments are carried out, and the experimental results prove the rationality of our architecture design. In summary, based on the SDNES architecture, a variety of drone services can be implemented, such as drone navigation, drone data real-time sharing, data distributed secure storage, and so on, all of which can assist and accelerate drone flying automation.

In addition to the above challenges, there are some open issues that should be considered in the future. They can be roughly divided into two aspects: software and hardware. In terms of software, for example, in order to achieve the fairness and accuracy of credit evaluation, it is necessary to choose reasonable evaluation criteria for specific services. Second, in order to avoid network jams caused by blockchain data broadcasting, what data are recorded in the blockchain should also be carefully considered. Moreover, as the information interactions between drones become more and more frequent, the security of the drone communication network is becoming increasingly prominent. Therefore, doing research on secure routing protocols and communication is also crucial [21,30]. Last but not least, other interesting topics include how to design reasonable incentive mechanisms to attract more high-performance drones to join the drone network to provide edge services, and whether it is more suitable to use the Consortium Blockchain [31] to meet the large number of data trades in drone flying automation. In terms of hardware, the first thing to consider is the drone battery life and the duration of each flight. At present, the battery capacity of drones is limited to about half an hour, which greatly limits the operation time and scope of drones. Therefore, how to achieve good power management, reduce energy consumption, and extend the duration of battery are urgent problems to be solved. Hardware facilities used to assist drone flights, such as sensors and radars, should also be further upgraded to make them lighter and more precise. To sum up, the realization of real drone flying automation requires more intelligent and safe software, as well as the support of hardware meeting specific standards.
